# Surveillance of SARS-CoV-2 virus circulation using Acute Respiratory Infections sentinel system of Catalonia (PIDIRAC) during the 2019-2020 season: A retrospective observational study

**DOI:** 10.1371/journal.pone.0264949

**Published:** 2022-03-14

**Authors:** Mireia Jané, Ana Martínez, Pilar Ciruela, Mar Mosquera, Miguel J. Martínez, Luca Basile, Mª José Vidal, Mª Mercè Nogueras, Patricia de Molina, Jordi Vila, Mª Angeles Marcos

**Affiliations:** 1 Public Health Agency of Catalonia (ASPCAT), Barcelona, Spain; 2 Epidemiology and Public Health CIBER, Instituto Carlos III, Madrid, Spain; 3 University of Barcelona, Barcelona, Spain; 4 Microbiology Laboratory, Hospital Clinic-ISGlobal, University of Barcelona, Barcelona, Spain; Centers for Disease Control and Prevention, UNITED STATES

## Abstract

**Background:**

In the context of COVID-19 pandemic in Catalonia (Spain), the present study analyses respiratory samples collected by the primary care network using Acute Respiratory Infections Sentinel Surveillance System (PIDIRAC) during the 2019–2020 season to complement the pandemic surveillance system in place to detect SARS-CoV-2. The aim of the study is to describe whether SARS-CoV-2 was circulating before the first confirmed case was detected in Catalonia, on February 25^th^, 2020.

**Methods:**

The study sample was made up of all samples collected by the PIDIRAC primary care network as part of the Influenza and Acute Respiratory Infections (ARI) surveillance system activities. The study on respiratory virus included coronavirus using multiple RT-PCR assays. All positive samples for human coronavirus were subsequently typed for HKU1, OC43, NL63, 229E. Every respiratory sample was frozen at—80°C and retrospectively studied for SARS-CoV-2 detection. A descriptive study was performed, analysing significant differences among variables related to SARS-CoV- 2 cases comparing with rest of coronaviruses cases through a bivariate study with Chi-squared test and statistical significance at 95%.

**Results:**

Between October 2019 and April 2020, 878 respiratory samples from patients with acute respiratory infection or influenza syndrome obtained by PIDIRAC were analysed. 51.9% tested positive for influenza virus, 48.1% for other respiratory viruses. SARS-CoV-2 was present in 6 samples. The first positive SARS-CoV-2 case had symptom onset on 2 March 2020. These 6 cases were 3 men and 3 women, aged between 25 and 50 years old. 67% had risk factors, none had previous travel history nor presented viral coinfection. All of them recovered favourably.

**Conclusion:**

Sentinel Surveillance PIDIRAC enhances global epidemiological surveillance by allowing confirmation of viral circulation and describes the epidemiology of generalized community respiratory viruses’ transmission in Catalonia. The system can provide an alert signal when identification of a virus is not achieved in order to take adequate preparedness measures.

## Introduction

On December 31^st^, 2019, the Wuhan Municipal Health Commission in the Province of Hubei, China, announced a cluster of 27 cases of pneumonia of unknown aetiology, including 7 severe cases, with a common exposure taking place in a wholesale market of seafood, fish and live animals in the city of Wuhan. No outbreak source was identified [1]. The market was shut down on January 1st, 2020. On January 7th, 2020, Chinese authorities identified a novel type of virus from the *Coronaviridae* family as the causative agent of the outbreak, which was called coronavirus SARS-CoV-2 [2]. The Chinese authorities shared its genetic sequence on January 12th, 2020. On January 30th, 2020, the World Health Organization declared the outbreak of coronavirus SARS-CoV-2 a public health emergency of international concern in China [3]. Following this, the outbreak spread beyond China, affecting other countries, many of them in Europe. In February, the number of cases started to fall in China as it increased in other countries, especially in Europe [4].

Reported cases increased dramatically and spread exponentially around the world. On March 11th, 2020, SARS-CoV-2 infection was declared as a pandemic by WHO [5]. The outbreak in Italy affected a large number of cases and, from there, spread to Spain. In Catalonia, a region in the northeast of Spain with 7.5 million inhabitants, as to May 27^th^, 2020, the number of confirmed cases was 58,480 [6, 7].

The first confirmed case of SARS-CoV-2 in Catalonia was notified on 25 February 2020 to the Catalonia Epidemiological Surveillance Network (XVEC). It was a 36-year-old female Barcelona resident who had travelled from 12 to 22 February to the cities of Bergamo and Milano. Symptoms appeared on 20 February; she required hospitalization and developed no complications.

Cases increased slowly in Catalonia during the containment first stage of the pandemic- as XVEC strictly applied the action protocol for the new coronavirus SARS-CoV-2 infection drawn up by the Surveillance and Public Health Emergency Response General Subdirectorate [8] agreed upon as part of the State Epidemiological Surveillance Network (RENAVE) and by the Coordination Centre for Sanitary Alerts and Emergencies (CCAES) [9]. An active surveillance of cases and contacts was necessary to establish the appropriate tests and to implement isolation and quarantine when required, which implied cases’ close contacts movement restriction. This was a pioneering implemented measure as Catalonia kept a limited transmission of the located chains for some time before reaching community transmission on March 14^th^, 2020.

In addition to the active surveillance system established as a response to the health alert, Catalonia had a sentinel surveillance system for influenza and other acute respiratory infections (PIDIRAC) implemented since the 1999–2000 season. It is based on a surveillance network made up of 56 primary health care physicians, and pediatricians who work in 44 primary care health centers homogenously distributed in Catalonia who report influenza like illnesses and other acute respiratory infections. The system combines syndromic sentinel surveillance and virological assessment of circulating respiratory viruses such as human influenza or respiratory syncytial virus. It has specific guidelines for case reporting, sample collection and other operational features that are assessed and reviewed for updates by Surveillance and Public Health Emergency Response General Subdirectorate staff before the onset of the seasonal surveillance period. The PIDIRAC system is part of the national influenza surveillance system. For many years [10, 11], it has been supported by several systems and information sources from different territories around the country, being also part of the European Influenza Surveillance Network (EISN) which is coordinated by the European Centre for Disease Control (ECDC) since 2008, contributing to the overall objectives in terms of influenza international surveillance. The EISN also encompases several laboratories that are part of the European Reference Laboratory Network for Human Influenza (ERLI-Net).

The objective of this study is to describe and to analyse the respiratory samples obtained in primary care as part of PIDIRAC to complement the active surveillance system implemented in the pandemic and to establish whether SARS-CoV-2 was circulating before the first case was detected in Catalonia, between October 2019 and April 2020 and could have been detected by PIDIRAC. This is important from the public health perspective.

## Methods

### Surveillance system

In January 2020, Catalonia deployed an enhanced surveillance system due to the public health emergency of international concern triggered by SARS-CoV-2, in collaboration with RENAVE and CCAES. During the containment stage, and following WHO guidelines, the clinical and epidemiological criteria of the action protocol for suspected cases of infection of the new coronavirus SARS-CoV-2 [8]was implemented. Clinical and epidemiological criteria changed with the evolution of the infection and the pandemic stage. Once the first local cases emerged in Italy on February 21^st^, 2020 [12], a case under investigation was any individual with a clinical condition compatible with acute respiratory infection of sudden onset and some of the following symptoms: cough, fever and dyspnea regardless of its severity. Also, suspected cases who, in the 14 days prior to symptoms onset, met any of the following epidemiological criteria: travel history to areas with evidence of community transmission, such as China (all provinces, including Hong Kong and Macao), South Korea, Iran, Japan, Singapore and Italy (especially Lombardy, Veneto, Emilia-Romagna, Piedmont). Cases under investigation were tested for the presence of SARS-CoV-2 in nasopharyngeal swabs using reverse transcription polymerase chain reaction assays (RT-PCR). Additionally, other cases outside these areas, with exposure to a cluster of cases, transmission chains of significant size and a history of close contact with a possible or confirmed case, were also investigated.

PIDIRAC continued its activity with professionals who collected pharyngeal and nasal samples from the beginning of the seasonal influenza surveillance on October 2019 until April 2020. Besides influenza A, B, and C virus, other virus under study included: respiratory syncytial virus, parainfluenza virus, adenovirus, coronavirus, enterovirus, rhinovirus, metapneumovirus and bocavirus. Sentinel physicians notified cases or cluster of cases presenting with Influenza Like Illness (ILI) or acute respiratory infection symptoms (ARI) on a daily basis to the Public Health Agency of Catalonia. Respiratory samples were systematically collected by nasopharyngeal swabbing according to the standardized protocol and sent for processing at the Hospital Clínic of Barcelona laboratory, a reference laboratory for respiratory virus in Catalonia. The coordination of the system is carried out by the Sub-directorate of Surveillance and Emergency Response of the Public Health Agency of Catalonia. The system has a quick communication email group which includes all participating sentinel physicians, and the epidemiology and microbiology teams involved. Weekly reports are deployed and uploaded to the Public Health Agency of Catalonia web page. It has specific guidelines for communication, sample collection and other operational features that are assessed and reviewed for updates by the Sud-Directorate public health staff before the onset of the seasonal surveillance period. Furthermore the system is in constant information flow with the National Influenza Surveillance System (SVGE) that in turn report to the European Centre for Disease Control and subsequently to the World Health Organization FluNet system.

### Microbiology

All samples collected by the PIDIRAC sentinel surveillance network were part the routine respiratory infection surveillance activities on influenza A, B and C virus, adenovirus, respiratory syncytial virus, bocavirus, metapneumovirus, parainfluenza virus, rhinovirus, enterovirus and coronavirus (CoV) using multiple RT-PCR assays.

The presence of respiratory viruses was analysed with a semi-automated platform (Flow system, Roche Diagnostics GmbH, Mannheim, Germany) that includes nucleic acid extraction with MagnaPure 96 and amplification and detection of the virus using a LightCycler 480. Specific primers and probes (LightMix^®^ Modular, TIB MOLBIOL, GmbH, Berlin, Germany, catalogue numbers: 58-0120-96, 50-0656-96, 58-0125-96, 50-0129-96, 50-0128-96, 61-0110-96, 64-0123-96, 53-0101-96, 58-0102-96, 64-0104-96) were used for the viruses mentioned above. All positive samples for coronavirus were subsequently typed for human coronavirus: HKU1, OC43, NL63, 229E. Every respiratory sample had been frozen at—80°C and retrospectively studied for the presence of SARS-CoV-2 with the Cobas SARS-CoV-2 assay on the 6800 platform (Roche Diagnostics). The Cobas 6800 assay can amplify and detect two viral targets: ORF1 a/b, a non-structural region that is unique to SARS-CoV-2 and a conserved region in the E-gene, which is a structural protein envelope for pan-Sarbecovirus detection [13].

### Statistical analysis

For each case sampled, the following variables were registered and analysed: age, sex, municipality, date of symptom onset, case diagnosis and notification, clinical presentation, risk factors (comorbidities), hospitalization, ICU admission, travel history, close contact of COVID19 and whether it was a health care professional. A descriptive study was performed and the presence of significant differences of these variables between SARS-CoV-2 cases and the rest of coronaviruses was analysed in a bivariate study by Chi-squared test, setting statistical significance at 95% confidence interval (CI).

### Ethical considerations

All data used in the analysis were collected during routine public health surveillance activities, as part of the legislated mandate of the Health Department of Catalonia, the competent authority for the surveillance of communicable diseases, which is officially authorized to receive, treat and temporarily store personal data on cases of infectious disease. Therefore, all study activities were part of the public surveillance and were, as a result, exempt from institutional board review and did not require informed consent. All data were fully anonymized.

## Results

**Fig 1:**
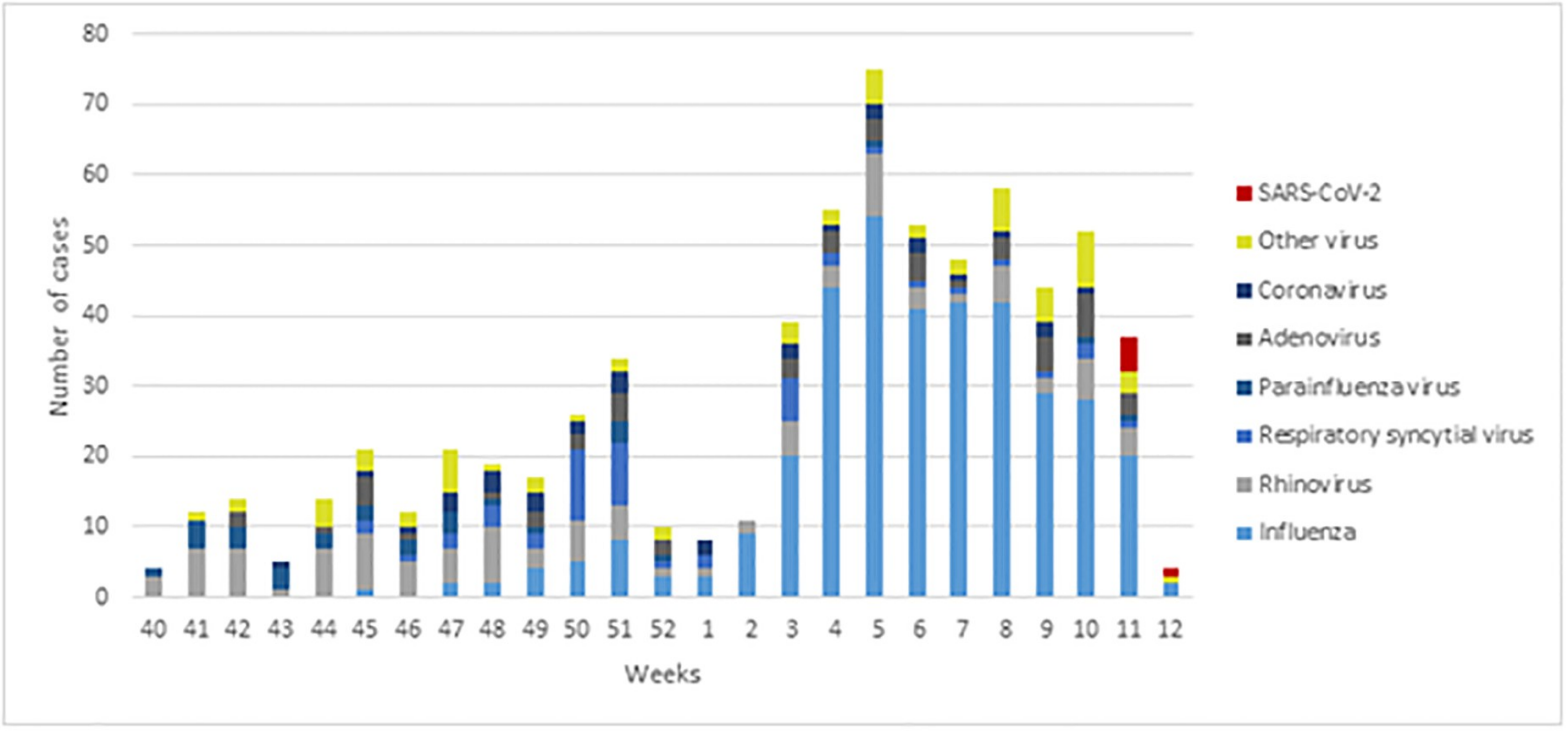
Respiratory virus in the population during winter season. Catalonia, October 2019-April 2020.

From October 1^st^, 2019, to April 1^st^, 2020, a total of 878 sentinel respiratory specimens from patients presenting influenza-like illness (ILI) or acute respiratory infection (ARI) symptoms were analysed as part of the PIDIRAC Program. Overall, 69.7% (612) tested positive for respiratory virus, with a total of 691 viral detections. Fig 1 shows the circulating virus during the winter season in Catalonia. Among the respiratory viruses detected, 51.9% were positive for influenza virus and 48.1% for another respiratory virus from which 5.1% (35) were CoV. Virus distribution is shown in Table 1.

**Table 1:** Results of detection due to virus. Samples from PIDIRAC sentinel network. 2019–2020 season.

Virology results	Total Season	
	N	%
**Influenza virus**	**359**	**51.9%**
Influenza A	163	23.6%
A(H1N1)pdm09	94	13.6%
A(H3N2)	58	8.4%
A no subtype	11	1.6%
Influenza B	194	28.1%
Influenza C	2	0.3%
**Other virus**	**332**	**48.0%**
Rhinovirus	107	15.5%
Adenovirus	50	7.2%
Respiratory syncytial virus	48	6.9%
Coronavirus	35	5.1%
Parainfluenza virus	29	4.2%
Bocavirus	29	4.2%
Metapneumovirus	17	2.5%
Enterovirus	17	2.5%
**Total detections**	**691**	**100.0%**

The 35 CoV viruses were typed as follows: 18 NL63, 5 HKU1, 5 OC43, 1 as 229E, while the remaining 6 were not typed. In the retrospective study for SARS-CoV-2, only 6 out of the 878 samples tested positive, coinciding with the 6 untyped samples positive to coronavirus. These 6 CoV tested positive to SARS-CoV-2 in the subsequent tests on the Cobas 6800.

The first SARS-CoV-2 positive case in the study had onset of symptoms on March 2^nd^, 2020, and the last one on March 16^th^, 2020. In the meantime, cases positive to other CoV, symptoms onset started between October 20^th^, 2019 and March 7^th^, 2020. In 4 cases (66.7%), patients with SARS-CoV-2 showed a sudden onset of symptoms while 12 patients (41.4%) affected by other coronavirus had a sudden onset of symptoms. Fever and dyspnea were more frequent in the SARS-CoV-2 cases (100% and 33.3%, respectively) than in the other CoV cases (72.4% and 6.9%, respectively), although without significant differences between the two groups. Comparison of symptoms between SARS CoV-2 with all other viruses and shown are included in Table 2.

**Table 2:** Clinical and epidemiological characteristics of SARS-CoV-2 and other CoV. Catalonia, October 2019-April 2020.

	SARS-CoV-2 n	%	Others CoV n	%	p
**Age group**					
1–9 years	-	-	8	27.6	
10–19 years	-	-	1	3.4	
20–29 years	1	16.7	4	13.8	NS
30–39 years	1	16.7	-	-	NS
40–49 years	3	50.0	7	24.1	NS
50–59 years	1	16.7	4	13.8	NS
60–69 years	-	-	2	6.9	
70–79 years	-	-	2	6.9	
80–89 years	-	-	1	3.4	
**Total**	**6**		**29**		
**Sex**					
Female	3	50.0	17	58.6	NS
Male	3	50.0	12	41.4	NS
**Total**	**6**		**29**		
**Clinical**					
Fever	6	100.0	21	72.4	NS
Cough	5	83.3	23	79.3	NS
Arthromyalgia	4	66.7	19	65.5	NS
Dyspnoea	2	33.3	2	6.9	NS
Headache	3	50.0	13	44.8	NS
Odynophagia	2	33.3	17	58.6	NS
General discomfort	6	100.0	24	82.7	NS
Chills	4	66.7	15	51.7	NS
**Risk factors**	1	16.7	-	-	NS
Immunodeficiency					
Chronic respiratory disease	2	33.3	6	20.7	NS
Cardiovascular disease	-	-	2	6.9	
Obesity	1	16.7	1	3.4	NS
Metabolic disease	-	-	3	10.3	
**Total**	**4**	**66.6**	**12**	**41.4**	NS
**Hospitalization**					
Yes	1	16.7	-	-	NS
**ICU admission**					
Yes	-	-	-	-	
**Death**					
Yes	-	-	-	-	
**Coinfection**					
Yes	0	0.0	8	27.6	NS

The SARS-CoV-2 patients were 3 men and 3 women, aged 25 to 50 years old with mean age of 44.5 years old and residents in the health regions of Girona (4 cases), Catalunya Central (1 case) and Barcelona Sud (1 case).

Patients identified as positive to other CoV were 17 women (58.6%) and 12 men (41.4%), aged 1 to 87 years old. Cases caused by other CoV infections (29 cases) were distributed as follows throughout Catalonia: 6 Barcelonès Nord, 3 Barcelonès Sud, 4 Barcelona city, 4 Catalunya Central, 8 in Tarragona, 1 in Girona, 1 Terres de l’Ebre, 1 in Lleida and 1 in Alt Pirineu-Aran.

In 4 cases (66.6%), SARS-CoV-2 patients suffered some underlying pathology that implied an increased risk of infection while in the other CoV cases, only 12 patients (41.4%) presented risk factors. None of the positive SARS-CoV-2 cases suffered from coinfection with another virus under study, while 8 of other positive CoV (27.6%) were coinfected with another respiratory virus: 2 with rhinovirus (HRV), 2 with respiratory syncytial virus (HRSV), 1 with influenza virus (FlubV), 1 bocavirus (HBoV), 1 adenovirus (HAdv) and 1 parainfluenza-2 virus (HPIV-2). None of the SARS-CoV-2 cases had been in contact with a possible case of influenza or SARS-CoV-2 nor did they have travel history to areas with community transmission at that time. One of the SARS-CoV-2 cases was a health care professional.

All patients recovered favourably. None of the cases affected with non-SARS-CoV-2 required hospitalization. One of the cases with SARS-CoV-2, with previous respiratory pathology, required hospitalization and recovered favourably.

## Discussion

During the study period, 878 respiratory samples were analysed. 51.9% tested positive for influenza virus and 48.1% for other respiratory viruses. SARS-CoV-2 was present in 6 samples.

In the case of a pandemic like SARS-CoV-2, combination of different epidemiological surveillance systems is essential to enhance monitoring and early detection of circulating viruses as much as possible in order to implement different public health actions as required depending on pandemic stage.

In Catalonia, the first COVID-19 case was detected on February 25th. From that moment until mid-March, transmission chains of the virus were perfectly located during the containment stage and cases were immediately studied and followed-up to contain the spread of the disease [8]. Through the data from the PIDIRAC system, functioning since October 2019, the study could ascertain that although the regular seasonal coronavirus was already circulating, generalised presence of SARS-CoV-2 in the community started at the beginning of March, and transmission increased from that moment onwards. Therefore, we conclude that the PIDIRAC system did not detect any case prior to the first XVEC notification because of a lack of SARS-CoV-2 generalized circulation within the community.

The first six SARS-CoV-2 cases reported through the PIDIRAC sentinel network were mild cases detected during the containment stage, after the first SARS-CoV-2 case was detected by XVEC.

Despite the absence of significant clinical differences between SARS-CoV-2 and the other human CoV, it seems that SARS-CoV-2 mainly affected adults with risk factors while endemic CoV affected almost all age groups and, to a lesser degree, patients with comorbidities. These results show the importance of microbiological surveillance as a key point to reinforce virus detection.

None of the SARS-CoV-2 cases had coinfection with another respiratory virus under study unlike endemic CoV cases which showed coinfection with a different respiratory virus (almost one third of the cases). Similarly, Gaunt ER et al. in UK describe coinfection rate of 11 to 41% [14] between CoV and other respiratory viruses such as RSV, influenza and adenovirus.

Following WHO recommendations, both surveillance systems sentinel and pandemic containment systems- coexisted in Catalonia. Likewise, European countries progressively implemented a strong surveillance of COVID-19 cases to draw a global strategy to contain COVID-19, which was progressively complemented with different epidemiological surveillance systems [3, 15]. The sentinel surveillance systems are recommended by the European surveillance network, especially in European regions or states where mild cases were not tested at the beginning, as it is our case [16].

France was the first European country to diagnose 3 cases on January 24^th^, 2020. These were people with a travel history to Wuhan [17]. Its acute respiratory infections sentinel surveillance system showed that several nasopharyngeal swabs tested positive to SARS-CoV-2. Excess of cases due to acute respiratory infection was quantified in France at the beginning of March compared to the number of visits expected from the seasonal influenza virus epidemic [18].

Regarding the study limitations, it is worth noting that the respiratory clinical portrait of influenza in the context of the PIDIRAC Program could not allow for identification of a COVID-19 possible case, either because symptoms were very mild and the person did not visit the physician or because it was an asymptomatic COVID-19 case. The presence of pathogens others than SARS-CoV-2 does not guarantee that a patient was not SARS-CoV-2 positive [19]. However, our study did not show any coinfection with another respiratory virus. Fabiana Gámbaro et al. demonstrates that the virus had been previously circulating in France, with the additional challenge of asymptomatic cases [20]. Similarly, as the sentinel surveillance system is focused on community, it would have not detected severe influenza syndrome cases that had been hospitalised prior to the announcement of the international public health emergency at the end of January. However, a strength of PIDIRAC system is that the surveillance started in October 2019, several months before the SARS-CoV-2 epidemic emergency declaration. In addition, we corroborated that the other regular Catalan epidemiological surveillance systems, i.e., the compulsory declaration of transmitted diseases system (MDO) and Catalonia microbiological notifications system (SNMC), did not detect any case before 25 February.

The system is robust enough to monitor most human respiratory virus into community and we have included SARS-CoV-2 sequence at laboratory, essential to study genomic evolution of the virus. Several years ago, the system was designed mainly for the surveillance of influenza and to detect genetic changes that could imply a pandemic threat for influenza. In that sense, a recommendation that derives from the study is that genetic analysis of untyped respiratory viruses detected by the PIDIRAC system could be included in a microbiological data bank as an alert.

The acute respiratory infections sentinel surveillance system, PIDIRAC, reinforced the global epidemiological surveillance, allowed for confirming whether the virus was circulating or not, and helped to verify that generalised community transmission in Catalonia took place in mid-March 2020 onwards. Public Health departments should rely on sentinel systems to determine SARS-CoV-2 community transmission levels, monitor molecular and epidemiologic changes and thus prioritise community mitigation measures [21]. Zwald ML et al. showed that COVID-19 case identification through a sentinel surveillance system helped to confirm SARS-CoV-2 community transmission and that the implementation of early community measures is key for a more effective mitigation of SARS-CoV-2 transmission [21, 22]. As we approached generalised SARS-CoV-2 circulation, surveillance based on clinical criteria and sample collection by sentinel physicians was essential to assess the situation [23]. PIDIRAC sentinel system is useful to monitor SARS-CoV-2 community transmission and geographic expansion, as it has done for seasonal influenza during decades. It is useful as well to alert and to be prepared in case of a potential emergency that may arise from a virus outbreak with pandemic potential, such as influenza, while the COVID-19 pandemic remains active [24]. Continuous system improvement to increase population coverage and improve its agility to obtain results and become more efficient is being permanently carried out. Our study shows that reinforcing epidemiological surveillance by using two different epidemiological systems that complement each other as well as improving microbiological surveillance is key to better understanding of the epidemiological pattern of respiratory viruses in Catalonia and increase detection capabilities.

To our knowledge, this is the first study in Catalonia that describes the presence or absence of SARS-CoV-2 detection in human respiratory samples previous to the first case detected on 25th February 2020, through a global Catalan sentinel surveillance system. This is important from the public health perspective in order to confirm the circulation of SARS-CoV-2 among the Catalan population and to highlight that a sentinel surveillance system is an important tool to monitor any type of respiratory virus circulation. In addition, PIDIRAC system is flexible in order to be adapted and prepared when SARS-CoV-2 virus becomes endemic since it has the advantage of being already in place, able to monitor incidence, epidemiological and microbiological characteristics of respiratory virus in a sustainable way and helps public health in the decision making process.

## References

[1] European Centre for Disease Prevention and Control. Cluster of pneumonia cases caused by a novel coronavirus, Wuhan, China;—17 January 2020. ECDC: Stockholm; 2020.

[2] European Centre for Disease Prevention and Control. Outbreak of severe acute respiratory syndrome coronavirus 2 (SARS-CoV-2): increased transmission beyond China − fourth update, 14 February 2020. ECDC: Stockholm; 2020.

[3] European Centre for Disease Prevention and Control. Outbreak of acute respiratory syndrome associated with a novel coronavirus, China: first local transmission in the EU/EEA—third update. 31 January 2020. ECDC: Stockholm; 2020.

[4] World Health Organization. (2020). Critical preparedness, readiness and response actions for COVID-19: interim guidance, 7 March 2020.

[5] European Centre for Disease Prevention and Control. Novel coronavirus disease 2019 (COVID-19) pandemic: increased transmission in the EU/EEA and the UK—sixth update– 12 March 2020. Stockholm: ECDC; 2020.

[6] Informe tècnic de resum dels casos de la COVID-19 a Catalunya. Número 12. [Technical summary report of COVID-19 cases in Catalonia. Number 12] Sub-direcció General de Vigilància i Resposta a Emergències de Salut Pública. Departament de Salut. [Deputy director of public health surveillance and response to emergencies. Department of Health].

[7] Nabil B, Sabrina B, Abdelhakim B. Transmission route and introduction of pandemic SARS-CoV-2 between China, Italy, and Spain. J Med Virol 2021;93:564–8. doi: 10.1002/jmv.26333 32697346 PMC7404595

[8] Procediment d’actuació enfront de casos d’infecció pel nou coronavirus SARS-CoV-2. Sub-direcció General de Vigilància i Resposta a Emergències en Salut Pública. Departament de Salut. [Deputy director of public health surveillance and response to emergencies. Department of Health].

[9] Procedimiento de actuación frente a casos de infección por el nuevo coronavirus (SARS-CoV-2). [Action protocol for infection cases by the novel coronavirus (SARS-CoV-2)] Centro de Coordinación de Alertas y Emergencias Sanitarias. Ministerio Sanidad. [Coordination Centre for Sanitary Alerts and Emergencies. Ministry of Health].

[10] Pla d’informació de les infeccions respiratòries agudes a Catalunya (PIDIRAC) 2019–2020. [Information Plan on acute respiratory diseases in Catalonia (PIDIRAC) 2019–2020] Sub-direcció General de Vigilància i Resposta a Emergències en Salut Pública. Departament de Salut. [Deputy director of public health surveillance and response to emergencies. Department of Health].

[11] Sistema de Vigilancia de la Gripe en España [Spanish Influenza Surveillance System] https://vgripe.isciii.es/inicio.do;jsessionid=0B3EE143B7F54A3A22B96759357097B2.

[12] Ciro Indolfi and Carmen Spaccarotella. The Outbreak of COVID-19 in Italy. Fighting the pandemic. JACC: Case Reports. April 2020. 10.1016/j.jaccas.2020.03.012 PMC7270641 32835287

[13] Poljak M, Korva M, Knap Gašper N, Fujs Komloš K, Sagadin M, Uršič T et al. Clinical evaluation of the cobas SARS-CoV-2 test and a diagnòstic platform switch during 48 hours in the midst of the COVID-19 pandemic. J Clin Microbiol 2020; 58 (6): e00599–20. doi: 10.1128/JCM.00599-20 32277022 PMC7269406

[14] Gaunt E. R., Hardie A., Claas E. C. J., Simmonds P., and Templeton K. E. Epidemiology and Clinical Presentations of the Four Human Coronaviruses 229E, HKU1, NL63, and OC43 Detected over 3 Years Using a Novel Multiplex Real-Time PCR Method. J Clin Microbiol. 2010; 48(8): 2940–2947. doi: 10.1128/JCM.00636-10 20554810 PMC2916580

[15] World Health Organization. Novel coronavirus (2019-nCoV). Situation report-11.https://www.who.int/docs/default-source/coronaviruse/situation-reports/20200131-sitrep-11-ncov.pdf?sfvrsn=de7c0f7_4.

[16] European Centre for Disease Prevention and Control. Strategies for the surveillance of COVID-19. Stockholm: ECDC; 2020.

[17] Bernard Stoecklin S, Rolland P, Silue Y, Mailles A, Campese C, Simondon A et al. First cases of coronavirus disease 2019 (COVID-19) in France: surveillance, investigations and control measures, January 2020. Euro Surveill. 2020; 25(6). doi: 10.2807/1560-7917.ES.2020.25.6.2000094 32070465 PMC7029452

[18] Boëlle Pierre-Yves, Souty Cécile, Launay Titouan, Guerrisi Caroline, Turbelin Clément, Behillil Sylvie, et al. Excess cases of influenza-like illnesses synchronous with coronavirus disease (COVID-19) epidemic, France, March 2020. Euro Surveill. 2020; 25(14):2000326. 10.2807/1560-7917.ES.2020.25.14.2000326 PMC7160441 32290901

[19] European Centre for Disease Prevention and Control. Disease background of COVID-19. https://www.ecdc.europa.eu/en/covid-19/latest-evidence.

[20] Fabiana Gámbaro Sylvie Behillil, Baidaliuk Artem, Donati Flora, Albert Mélanie, Alexandru Andreea et al. Introductions and early spread of SARS-CoV-2 in France. BioRXiv. April, 2020. doi: 10.2807/1560-7917.ES.2020.25.26.2001200 32643599 PMC7346363

[21] Zwald ML, Lin W, Sondermeyer Cooksey GL, et al. Rapid Sentinel Surveillance for COVID-19- Santa Clara County, California. March 2020. MMWR Morb Mortal Wkly Rep 2020; 69:419–421. doi: 10.15585/mmwr.mm6914e3 32271724 PMC7147906

[22] Qualls N, Levitt A, Kanade N, et al.; CDC Community Mitigation Guidelines Work Group. Community mitigation guidelines to prevent pandemic influenza—United States, 2017.MMWR Recomm Rep 2017; 66 (1). doi: 10.15585/mmwr.rr6601a1 28426646 PMC5837128

[23] Spiteri G, Fielding J, Diercke M, Campese C, Enouf V, Gaymard A, et al. First cases of coronavirus disease 2019 (COVID-19) in the WHO European Region, 24 January to 21 February 2020. Euro Surveill. 2020 Mar; 25(9). doi: 10.2807/1560-7917.ES.2020.25.9.2000178 32156327 PMC7068164

[24] World Health Organization. Preparing GISRS for the upcoming influenza seasons during the COVID-19 pandemic—practical considerations. Interim guidance. May 2020.

